# Prognostic significance of neutrophil-to-lymphocyte and platelet-to-lymphocyte ratios in hepatocellular carcinoma: a retrospective multicenter study on overall and recurrence-free survival following liver resection or liver transplantation

**DOI:** 10.3389/fimmu.2026.1801659

**Published:** 2026-04-28

**Authors:** Konstantinos Arvanitakis, Spyridon Pantzios, Chrysanthos Christou, Vasileios Papadopoulos, Eleni Theocharidou, Christos Topalidis, Georgios Kalopitas, Orestis Sidiropoulos, Antonios Chatzigeorgiou, Ioannis Mitroulis, Triantafyllia Koletsa, Prodromos Hytiroglou, Georgios Katsanos, Ioannis Elefsiniotis, Georgios Tsoulfas, Georgios Germanidis

**Affiliations:** 1 Division of Gastroenterology and Hepatology, First Department of Internal Medicine, AHEPA University Hospital, Aristotle University of Thessaloniki, Thessaloniki, Greece; 2 Academic Department of Internal Medicine-Hepatogastroenterology Unit, General Oncology Hospital of Kifissia, National and Kapodistrian University of Athens, Athens, Greece; 3 Department of Transplantation Surgery, Hippokration General Hospital, School of Medicine, Aristotle University of Thessaloniki, Thessaloniki, Greece; 4 Laboratory of Anatomy, Medical School, Democritus University of Thrace, Alexandroupolis, Greece; 5 Second Department of Internal Medicine, Hippokration General Hospital, Aristotle University of Thessaloniki, Thessaloniki, Greece; 6 Department of Pathology, School of Medicine, Aristotle University of Thessaloniki, Thessaloniki, Greece; 7 Department of Physiology, Medical School, National and Kapodistrian University of Athens, Athens, Greece; 8 First Department of Internal Medicine, University Hospital of Alexandroupolis, Democritus University of Thrace, Alexandroupolis, Greece

**Keywords:** hepatocellular carcinoma, neutrophil-to-lymphocyte ratio, overall survival, platelet-to-lymphocyte ratio, prognostic biomarkers, recurrence-free survival, systemic inflammation

## Abstract

**Background:**

Systemic inflammation plays a critical role in hepatocellular carcinoma (HCC) progression and postoperative outcomes. This study assessed the prognostic value of the neutrophil-to-lymphocyte ratio (NLR) and platelet-to-lymphocyte ratio (PLR) for overall (OS) and recurrence-free survival (RFS) in patients undergoing curative hepatectomy or orthotopic liver transplantation (OLT).

**Methods:**

In this multicenter retrospective cohort of 74 HCC patients (86.5% male; median age 68.0 years), NLR and PLR were evaluated preoperatively and at 1-, 3-, 6-, and 12-months post-surgery. Associations with OS and RFS were evaluated using univariate, multivariable, and Cox proportional hazards models with time-varying covariates, while model discrimination was assessed using Harrell’s concordance index.

**Results:**

Elevated preoperative PLR predicted major postoperative complications (AUROC = 0.667, p = 0.030). The 3-month NLR demonstrated strong discriminative performance for OS (C-index 0.79). In time-varying Cox models, postoperative NLR emerged as a robust, time-independent predictor of OS (HR 1.35; p = 0.033), independent of the surgical procedure and Barcelona Clinic Liver Cancer stage. Multivariable analyses demonstrated that elevated NLR at 3-, 6-, and 12-months and PLR at 12 months independently predicted reduced OS. Hepatectomy was the dominant determinant of reduced RFS compared with OLT (p < 0.001), while an NLR threshold >2.5 identified a subgroup at markedly increased mortality risk, particularly following hepatectomy.

**Conclusions:**

Postoperative NLR is a powerful, time-independent prognostic biomarker for OS in surgically treated HCC, especially after liver resection, while PLR provides complementary prognostic information. These findings support the clinical integration of inflammation-based biomarkers into postoperative risk stratification and surveillance strategies.

## 1 Introduction

Primary liver cancer ranks as the third leading cause of cancer-related death and the sixth most diagnosed cancer worldwide. Globally, approximately 906,000 new cases of liver cancer and 830,000 related deaths are reported each year, reflecting an almost 1:1 ratio of incidence to mortality (1, 2). In high-income countries, alcohol use, chronic HCV infection, and metabolic dysfunction-associated steatotic liver disease (MASLD) are the major contributors to hepatocellular carcinoma (HCC), whereas chronic HBV infection remains the leading cause in developing regions (3). Men experience a two- to threefold greater disease burden than women, largely due to hormonal influences. Nonetheless, in some populations, women show increased susceptibility to HCV- and metabolic dysfunction-associated steatohepatitis (MASH)-related liver cancer (4). Despite improvements in surveillance and treatment, the prognosis remains poor due to high recurrence rates even after curative therapy (5). Therapeutic strategies for HCC should be individualized, as treatment options often overlap depending on patient-specific factors, while optimal disease management necessitates the collaboration of multiple specialists, underscoring the importance of a coordinated multidisciplinary team (MDT) approach (6). Liver resection (LR) and liver transplantation (LT) are the primary surgical treatments for HCC, both offering curative potential, but LT remains the only intervention capable of eradicating both the tumor and the underlying chronic liver disease (7). However, given its technical complexity, liver surgery demands a high level of expertise and should be performed in specialized centers with established proficiency in hepatobiliary surgery (8). Nonetheless, long-term outcomes vary widely, and simple prognostic biomarkers for stratification remain an unmet clinical need (9).

Chronic inflammation is being increasingly recognized as a hallmark of cancer initiation, progression, and treatment response across various malignancies (10). In the context of HCC, chronic hepatic injury (from viral hepatitis, alcohol use, metabolic syndrome) leads to repeated rounds of hepatocyte death and regeneration, inflammation, fibrosis, and ultimately tumorigenesis (11). The tumor microenvironment in HCC features pro-inflammatory cells, neutrophils and platelets, which can promote angiogenesis, tumor-cell proliferation, and metastasis, while lymphocytes mediate anti-tumor immunity (12, 13). The rapid expansion of therapeutic agents with diverse mechanisms of action in HCC underscores the importance of comprehensive molecular profiling, biomarker discovery, and robust treatment-response databases to enable precision therapy (14). Among emerging biomarkers, inflammatory indices such as the neutrophil-to-lymphocyte ratio (NLR) and platelet-to-lymphocyte ratio (PLR) have gained attention as accessible indicators of tumor biology, immune status, and systemic inflammation and they have emerged as potential prognostic biomarkers in several solid tumors, including HCC (15). As objective reflections of disease activity, these parameters hold promise for refining risk stratification, guiding treatment selection, and monitoring therapeutic response in HCC (16).

However, while numerous studies have explored the prognostic relevance of NLR and PLR in advanced or non-surgical settings and have reported associations of high NLR or high PLR with poorer survival in HCC, evidence regarding their longitudinal behavior and prognostic utility following curative-intent surgery remains limited (17, 18). Given the high recurrence rates and variable outcomes after LR or LT, identifying reliable, inexpensive, and easily measurable biomarkers is crucial. This study therefore aimed to evaluate the prognostic significance of NLR and PLR in predicting overall survival (OS) and recurrence-free survival (RFS) in patients with HCC undergoing curative hepatectomy or orthotopic liver transplantation.

## 2 Materials and methods

### 2.1 Study design and patient population

This retrospective multicenter cohort study included patients from three tertiary Greek hepatology/transplantation units: the First Department of Internal Medicine, AHEPA University Hospital (Aristotle University of Thessaloniki, Thessaloniki), the General Oncology Hospital of Kifissia (National & Kapodistrian University of Athens, Athens), and the Department of Transplantation Surgery, Hippokration General Hospital (Aristotle University of Thessaloniki, Thessaloniki). Approval of the study protocol has been obtained from the Institutional Review Board (IRB) and the Ethics Committee (No.: 3.683/18-1–2022 and 17285/11-4-2023), which granted a waiver of informed consent, as all data were retrospectively collected from patients’ medical records in an anonymized manner with no risk of personal data identification. The study was performed in accordance with the STROBE (Strengthening the Reporting of Observational Studies in Epidemiology) guidelines for observational studies and complied with the principles of the Declaration of Helsinki and its subsequent amendments (19, 20).

Patients identified from institutional databases that were treated in the aforementioned departments between January 2013 and December 2024 were screened for eligibility. Eligible participants included adult patients (≥18 years old) with a diagnosis of HCC established based on either liver biopsy and/or on the Liver Imaging Reporting and Data System (LI-RADS) developed by the American College of Radiology (21), who underwent surgical treatment with curative intent, including LR or orthotopic LT, with available of baseline and postoperative complete blood count (CBC) including neutrophils, lymphocytes and platelets at defined timepoints (pre-surgery and at 1, 3, 6 and/or 12 months). All patients were staged using the Barcelona Clinic Liver Cancer (BCLC) staging system (22). Patients were excluded if they belonged to the pediatric population (younger than 18 years of age) or if they underwent non-curative surgical procedures (R2 or palliative). Moreover, excluded from our study were patients with concurrent extra-hepatic malignancy and patients with missing key laboratory or follow-up data. Patients with postoperative death within 30 days were excluded to minimize perioperative mortality bias and ensure that survival analyses reflect long-term oncologic and inflammatory outcomes rather than early postoperative complications.

### 2.2 Data collection and variables

Baseline demographic, clinical and laboratory data were abstracted from electronic medical records. Collected variables included age, sex, body mass index (BMI), smoking status, alcohol consumption, comorbidities, etiology of underlying liver disease, Model for End-stage Liver Disease (MELD)-Na score and BCLC stage at diagnosis (0, A, B, C, D), tumor number of lesions and size, perioperative parameters (type of surgery: LR or orthotopic LT), and 30-day postoperative mortality. Complete blood counts were recorded at predefined time points: preoperatively (baseline) and postoperatively at 1, 3, 6, and 12 months. The NLR was calculated as the absolute neutrophil count divided by the absolute lymphocyte count, while the PLR was calculated as the platelet count divided by the absolute lymphocyte count. When multiple measurements were available within ±2 weeks of a target time point, the value closest to the scheduled time was selected.

### 2.3 Outcomes and statistical analysis

The primary endpoints were the OS and RFS. The OS was defined as the time from surgery (LR or orthotopic LT) to death from any cause or last follow-up. RFS was defined as time from surgery to radiologic/biologic evidence of HCC recurrence or death. For OS analyses, death from any cause was defined as the event and patients alive at last follow-up were censored, whereas for RFS, tumor recurrence was additionally treated as an event. Continuous variables are presented as mean ± standard deviation (SD) or median (interquartile range [IQR]) as appropriate, based on normality check using Kolmogorov-Smirnov and Shapiro-Wilk tests, while categorical variables are presented as counts and percentages. Differences between groups (LR vs orthotopic LT) were assessed using Student’s t-test or Mann-Whitney U-test for continuous variables and χ² or Fisher’s exact test for categorical variables. To assess the longitudinal and time-independent prognostic effect of NLR and PLR on survival outcomes, Cox proportional hazards models incorporating time-varying covariates were constructed for both OS and RFS. This approach was selected because NLR and PLR are dynamic biomarkers that change over time following surgery; thus, conventional Cox models based on a single measurement may introduce misclassification bias and fail to capture longitudinal biological effects of systemic inflammation. Time-varying models allow incorporation of serial measurements and estimation of the effect of biomarker trajectories on survival outcomes. In the primary analyses, NLR and PLR were modeled as continuous variables to preserve their full quantitative information. For clinical interpretability, additional analyses were performed using a categorical NLR cutoff (>2.5), derived from 3-month postoperative values using a 5-year survival discrimination approach. As this cutoff is data-driven, it should be considered exploratory and hypothesis-generating. Survival analyses were restricted to patients surviving beyond the initial 30-day postoperative period. Model discrimination for overall survival was assessed using Harrell’s concordance index (C-index). Internal validation was performed using 1,000 bootstrap resamples to estimate bias-corrected 95% confidence intervals for the C-index. Survival analyses were performed using Cox proportional hazards regression.

Initially, univariate Cox models were constructed to assess the association of NLR and PLR at predefined postoperative time points (baseline, 1, 3, 6, and 12 months) with OS and RFS. Multivariable Cox regression models were subsequently fitted to identify independent predictors of OS and RFS, adjusting for surgical procedure (hepatectomy vs transplantation) and Barcelona Clinic Liver Cancer (BCLC) stage (0/A vs B/C). To mitigate immortal-time bias inherent to postoperative biomarkers, a 3-month post-surgery landmark analysis was performed, restricting the cohort to patients alive and event-free at that time and measuring subsequent outcomes from the landmark forward. To explore residual confounding within the hepatectomy subgroup, sensitivity analyses were performed by adding, individually, vascular invasion, nodal spread, and prior transarterial chemoembolization (TACE) to the adjusted 3-month NLR model. Results are reported as hazard ratios (HRs) with 95% confidence intervals (CIs). Model performance was assessed using the corrected Akaike Information Criterion (AICc), calculated as: AICc = -2 log likelihood + 2k + [2k(k+1)]/(n - k - 1), where n is the sample size and k the number of parameters; when n/k>40, the small-sample correction term was neglected. AIC/AICc comparisons were restricted to models estimated on identical analysis sets; thus, NLR versus PLR models were compared within each time point, but not across time points. Statistical significance was defined as p < 0.05. Analyses were conducted using SPSS version 29.0 (IBM, Armonk, NY, USA) and STATA version 19.0 (StataCorp LLC, College Station, TX, USA). Graphical visualization of survival curves and model outputs was performed using STATA version 19.0 and R statistical software version 4.0.2 (R Foundation for Statistical Computing, Vienna, Austria).

## 3 Results

### 3.1 Baseline characteristics

The study cohort comprised 74 patients with HCC undergoing curative-intent surgery, of whom 64 (86.5%) were male. The median age of the overall population was 68.0 years (IQR 14.3), with forty-eight patients (64.9%) undergoing liver resection, while 26 (35.1%) met the Milan criteria and underwent orthotopic liver transplantation (8). Significant differences were observed between the hepatectomy and transplantation groups. Patients undergoing hepatectomy were significantly older, with a median age of 72.5 years (IQR 15.0), compared with 64.5 years (IQR 11.0) in the transplantation group (p = 0.011). No significant differences were observed between groups regarding sex distribution (p = 0.715), body mass index (p = 0.138), smoking status (p = 0.504), or alcohol consumption patterns (p = 0.279). Comorbid conditions were significantly more prevalent among patients treated with hepatectomy (81.3%) compared with those undergoing transplantation (50.0%, p = 0.005), reflecting differences in baseline clinical profiles and surgical candidacy. Liver disease severity also differed markedly between the two surgical modalities. Transplanted patients had significantly higher MELD-Na scores (median 9.4 [IQR 5.5] vs 7.0 [IQR 1.0]; p < 0.001) and higher Child-Turcotte-Pugh (CTP) scores (median 6.0 [IQR 3.0] vs 5.0 [IQR 0.0]; p < 0.001), consistent with more advanced underlying liver dysfunction in this group.

Regarding HCC etiology, chronic hepatitis B virus (HBV) infection was significantly more frequent among transplanted patients (61.5% vs 35.4%; p = 0.031), while metabolic dysfunction-associated steatohepatitis (MASH) was more common in the hepatectomy group, although this difference did not reach statistical significance (35.4% vs 15.4%; p = 0.068). The distribution of hepatitis C virus (HCV) infection was similar between groups (p = 0.387). Tumor-related characteristics also differed significantly. Patients undergoing hepatectomy had larger primary HCC lesions (median size 5.3 cm [IQR 5.8]) compared with transplanted patients (3.4 cm [IQR 2.1]; p < 0.001). Vascular invasion was present exclusively in the hepatectomy group (41.7% vs 0%; p < 0.001), as was nodal spread (20.8% vs 0%; p = 0.012). No patients had extrahepatic metastases at baseline. Despite these differences, the distribution of BCLC stages did not differ significantly between groups (p = 0.101), although advanced HCC stages (BCLC C) were observed only in the hepatectomy cohort. Early postoperative outcomes also differed substantially. Thirty-day postoperative morbidity occurred in 48.6% of the overall cohort and was significantly more frequent after transplantation (96.2%) compared with hepatectomy (22.9%; p < 0.001). Similarly, 30-day mortality was significantly higher among transplanted patients (23.1%) compared with none in the hepatectomy group (p = 0.001). Although 30-day postoperative mortality was recorded for descriptive purposes, these patients were excluded from subsequent survival analyses as predefined in the study design. Severe postoperative complications, as reflected by higher Clavien–Dindo grades, were also significantly more common in the transplantation group (p < 0.001).

Baseline inflammatory indices showed selective differences. Baseline NLR did not differ significantly between groups (p = 0.542). In contrast, baseline PLR was significantly higher in the hepatectomy group (median 107 [IQR 66]) compared with transplanted patients (median 71 [IQR 63]; p = 0.018). Postoperatively, NLR at 1 month was significantly higher in transplanted patients (median 4.7 [IQR 5.1] vs 2.3 [IQR 1.8]; p = 0.002), whereas no significant between-group differences were observed at later time points. Finally, RFS differed markedly by surgical modality, with significantly longer RFS observed in transplanted patients (mean 117.7 months, 95% CI 108.6-126.8) compared with those undergoing hepatectomy (mean 58.3 months, 95% CI 41.8-74.8; p < 0.001). Overall survival did not differ significantly between groups (p = 0.474), although transplanted patients had a significantly longer follow-up duration (mean 90.9 vs 54.4 months; p = 0.005). Detailed baseline demographic, clinical, tumor-related, and laboratory characteristics according to surgical modality are summarized in **Table 1**.

**Table 1:** Baseline demographic, clinical, tumor-related, perioperative, and inflammatory characteristics of patients with HCC undergoing curative-intent hepatectomy or orthotopic liver transplantation.

Parameter	All (n=74)	Hepatectomy (n=48)	Transplantation (n=26)	p-value
Sex				0.715
Females	10/74 (13.5)	7/48 (14.6)	3/26 (11.5)	
Males	64/74 (86.5)	41/48 (85.4)	23/26 (88.5)	
Age				**0.011**
Median [IQR]	68.0 [14.3]	72.5 [15.0]	64.5 [11.0]	
BMI				0.138
Mean [SD]	27.2 [4.0]	27.0 [4.3]	27.7 [3.3]	
Smoking				0.504
Current smoker	25/74 (33.8)	14/48 (29.2)	11/26 (42.3)	
Never smoked	38/74 (51.4)	26/48 (54.2)	12/26 (46.2)	
Ex-smoker	11/74 (14.9)	8/48 (16.7)	3/26 (11.5)	
Alcohol intake				0.279‡
Current overuse	1/74 (1.4)	1/48 (2.1)	0/26 (0.0)	
Mild use	4/74 (5.4)	4/48 (8.3)	0/26 (0.0)	
Never used	55/74 (74.3)	36/48 (75.0)	19/26 (73.1)	
Past overuse	14/74 (18.9)	7/48 (14.6)	7/26 (26.9)	
Comorbidities				**0.005**
No	22/74 (29.7)	9/48 (18.8)	13/26 (50.0)	
Yes	52/74 (70.3)	29/48 (81.3)	13/26 (50.0)	
MELD-Na score				**<0.001**
Median [IQR]	7.9 [2.4]	7.0 [1.0]	9.4 [5.5]	
CTP score				**<0.001**
Median [IQR]	5.0 [1.0]	5.0 [0.0]	6.0 [3.0]	
Etiology†				
HBV	33/74 (44.6)	17/48 (35.4)	16/26 (61.5)	**0.031**
MASH	21/74 (28.4)	17/48 (35.4)	4/26 (15.4)	0.068
HCV	9/74 (12.2)	7/48 (14.6)	2/26 (7.7)	0.387
Tumor size (main)				**<0.001**
Median [IQR]	4.2 [3.8]	5.3 [5.8]	3.4 [2.1]	
Vascular Invasion				**<0.001**
No	54/74 (73.0)	28/48 (58.3)	26/26 (100.0)	
Yes	20/74 (27.0)	20/48 (41.7)	0/26 (0.0)	
Nodal Spread				**0.012**
No	64/74 (86.5)	38/48 (79.2)	26/26 (100.0)	
Yes	10/74 (13.5)	10/48 (20.8)	0/26 (0.0)	
Extrahepatic metastases				**NA**
No	74/74 (100.0)	48/48 (100.0)	26/26 (100.0)	
Yes	0/74 (0.0)	0/48 (0.0)	0/26 (0.0)	
BCLC				0.101
0	2/74 (2.7)	1/48 (2.1)	1/26 (3.8)	
A	46/74 (62.2)	29/48 (60.4)	17/26 (65.4)	
B	17/74 (23.0)	11/48 (22.9)	6/26 (23.1)	
C	7/74 (9.5)	7/48 (14.6)	0/26 (0.0)	
D	2/74 (2.7)	0/48 (0.0)	2/26 (7.7)	
30d-morbidity				**<0.001**
No	38/74 (51.4)	37/48 (77.1)	1/26 (3.8)	
Yes	36/74 (48.6)	11/48 (22.9)	25/26 (96.2)	
30d-mortality				**0.001‡**
No	68/74 (91.9)	48/48 (100.0)	20/26 (76.9)	
Yes	6/74 (8.1)	0/48 (0.0)	6/26 (23.1)	
Clavien-Dindo				**<0.001**
0	37/74 (50.0)	36/48 (48.6)	1/26 (3.8)	
I	2/74 (2.7)	1/48 (2.1)	1/26 (3.8)	
II	20/74 (27.0)	10/48 (20.8)	10/26 (38.5)	
IIIa	3/74 (4.1)	1/48 (2.1)	2/26 (7.7)	
IIIb	5/74 (6.8)	0/48 (0.0)	5/26 (19.2)	
IVa	1/74 (1.4)	0/48 (0.0)	1/26 (3.8)	
IVb	0/74 (0.0)	0/48 (0.0)	0/26 (0.0)	
V	6/74 (8.1)	0/48 (0.0)	6/26 (26.0)	
NLR (baseline)				0.542
Median [IQR]	2.5 [2.1]	2.3 [1.5]	3.4 [2.8]	
NLR (1 month)				**0.002**
Median [IQR]	2.8 [3.1]	2.3 [1.8]	4.7 [5.1]	
NLR (3 month)				0.619
Median [IQR]	2.0 [1.8]	2.0 [2.0]	1.8 [1.7]	
NLR (6 month)				0.710
Median [IQR]	2.1 [1.7]	1.9 [1.9]	2.3 [1.7]	
NLR (12 month)				0.511
Median [IQR]	2.3 [1.2]	2.4 [1.5]	2.1 [0.9]	
PLR (baseline)				**0.018**
Median [IQR]	104 [64]	107 [66]	71 [63]	
PLR (1 month)				0.062
Median [IQR]	132 [108]	121 [84]	151 [143]	
PLR (3 month)				0.175
Median [IQR]	93 [57]	94 [72]	86 [40]	
PLR (6 month)				0.410
Median [IQR]	106 [49]	104 [51]	116 [60]	
PLR (12 month)				0.806
Median [IQR]	107 [44]	104 [50]	111 [43]	
RFS				**<0.001**
Mean (95% CI)	78.9 (65.7-92.2)	58.3 (41.8-74.8)	117.7 (108.6-126.8)	
OS				0.474
Mean (95% CI)	81.5 (69.1-93.9)	75.3 (58.2-92.4)	84.8 (62.3-105.3)	
Follow-up period				**0.005**
Mean (95% CI)	67.2 (58.0-76.4)	54.4 (43.4-65.4)	90.9 (80.9-100.8)	

MELD, model for end-stage liver disease; CTP, Child-Turcotte-Pugh; OS, overall survival; RFS, recurrence-free survival; IQR, interquartile range; CI, confidence interval; SD, standard deviation; NLR, neutrophil-to-lymphocyte ratio; PLR, platelet-to-lymphocyte ratio; BMI, body mass index; BCLC, Barcelona Clinic Liver Cancer; NA, Not Applicable.

†Two cases of HBV/HCV co-infection were observed.

‡Fisher’s exact test.

Bold values demonstrate statistical significance.

### 3.2 Prognostic performance of NLR as a time-varying covariate

To assess the overall and time-independent prognostic capacity of NLR, Cox proportional hazards models incorporating time-varying covariates were constructed for both OS and RFS. These models estimated the association between NLR and the hazard of the outcome over follow-up, adjusting for surgical procedure (transplantation vs hepatectomy) and BCLC stage (0/A vs B/C). This approach allowed the effect of NLR on the hazard to be evaluated as a function of time, prior to examining estimates at specific postoperative time points.

In the time-varying Cox regression model for OS (57 patients, 18 deaths, total time at risk 2,903.6 months), NLR remained a statistically significant independent predictor of mortality over time. Specifically, increasing NLR was associated with a higher risk of death (HR 1.348, 95% CI 1.024-1.773, p = 0.033). Importantly, this association persisted independently of surgical procedure and tumor stage. Hepatectomy (compared with transplantation) was associated with a significantly increased mortality risk (HR 3.63, 95% CI 1.26-10.42, p = 0.017), while early-stage disease (BCLC 0/A vs B/C) was protective (HR 0.22, 95% CI 0.06-0.82, p = 0.025). No significant interaction was observed between NLR and time, indicating that the prognostic effect of NLR on the OS was stable across follow-up. The overall model demonstrated strong discriminatory performance (likelihood ratio χ² = 21.52, p < 0.001). Model discrimination for overall survival was assessed using Harrell’s concordance statistic. The NLR at 3 months demonstrated good discriminative ability and predictive accuracy for survival outcomes, with a C-index of 0.791. Bootstrapped validation using 1,000 resamples yielded a stable estimate with a 95% CI 0.7577 - 0.8241, corresponding to discrimination significantly greater than 0.5 (p < 0.001) and thus, supporting the robustness of the model.

In contrast, when NLR was analyzed as a time-varying covariate in the RFS model, it did not retain statistical significance over time. Although surgical procedure and BCLC stage remained strong independent predictors of recurrence, the longitudinal effect of NLR on RFS was not significant after adjustment. Specifically, while early-stage disease (BCLC 0/A) was associated with a markedly reduced risk of recurrence (HR 0.30, 95% CI 0.19-0.47, p < 0.001) and transplantation conferred a protective effect (HR 0.50, 95% CI 0.30-0.85, p = 0.011), time-varying NLR showed no independent association with RFS (interaction with time: p = 0.995). This finding indicates that NLR does not exert a stable longitudinal effect on recurrence risk when modeled dynamically, despite associations observed at specific postoperative time points.

Collectively, these analyses demonstrate that NLR possesses robust, time-independent prognostic value for OS, irrespective of surgical modality, disease stage, or timing of measurement. Conversely, NLR does not exhibit stable longitudinal prognostic significance for RFS, suggesting that its impact is more closely related to survival after recurrence or non-recurrence-related mortality rather than recurrence risk itself. Based on these findings, subsequent analyses focused on specific postoperative time points, with particular emphasis on the 3-month postoperative NLR, which demonstrated strong discriminatory ability and was further evaluated using a 5-year survival horizon to define clinically meaningful cutoff values.

### 3.3 Temporal trends of NLR and PLR

**Figure 1:**
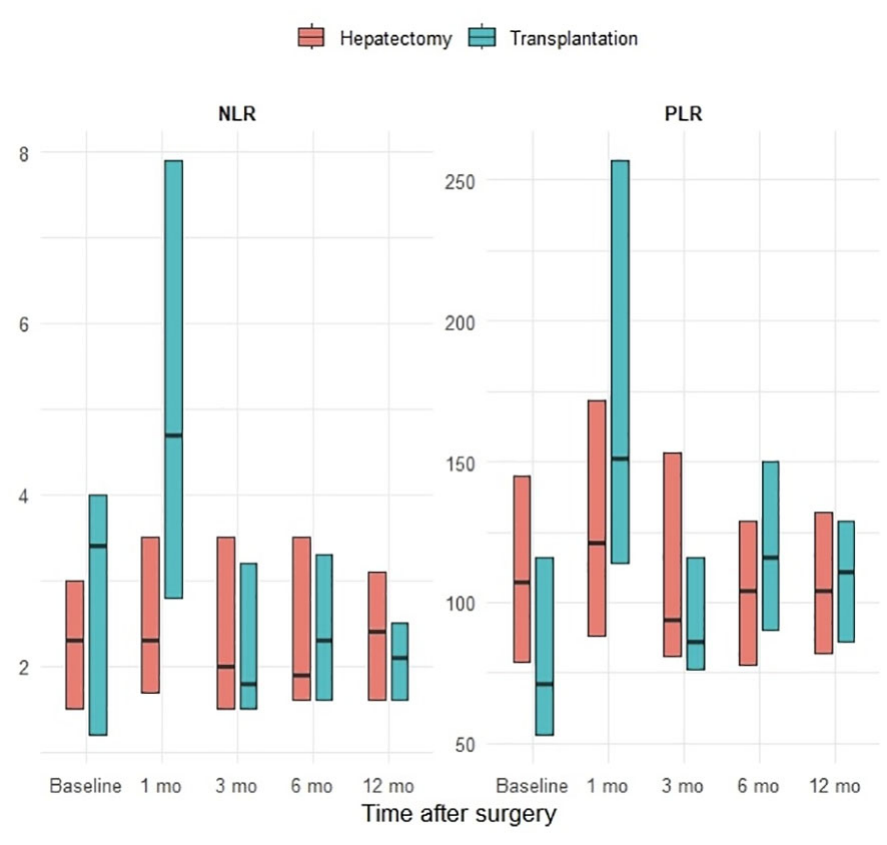
Longitudinal trends of neutrophil-to-lymphocyte ratio (NLR) and platelet-to-lymphocyte ratio (PLR) over the 12-month postoperative period. Values are presented as medians with interquartile range. Repeated-measures ANOVA demonstrated a significant temporal effect for PLR but not for NLR.

Longitudinal assessment demonstrated distinct postoperative patterns for NLR and PLR. Repeated-measures ANOVA showed that PLR changed significantly over the 12-month postoperative period (overall time effect p < 0.001), whereas NLR did not exhibit a statistically significant temporal variation (p = 0.182). These trends were more pronounced in the hepatectomy subgroup, while transplanted patients exhibited greater variability and less consistent longitudinal changes. Receiver operating characteristic analysis demonstrated that baseline PLR significantly predicted major postoperative complications (Clavien–Dindo grade ≥ III), with an AUROC of 0.667 (95% CI: 0.516-0.817; p = 0.030), whereas baseline NLR showed no significant predictive ability for severe complications. The longitudinal behavior of NLR and PLR is depicted in **Figure 1**.

### 3.4 Overall survival analysis

**Figure 2:**
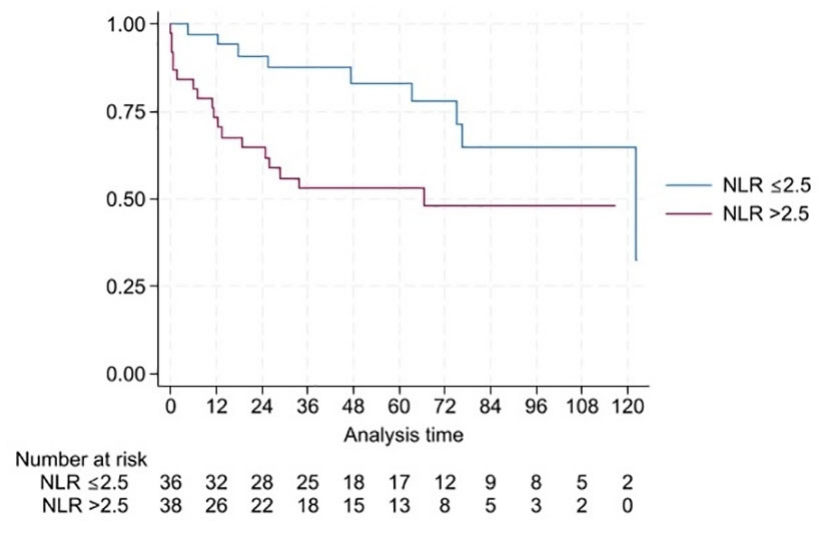
Kaplan-Meier curves for overall survival stratified by neutrophil-to-lymphocyte ratio (NLR) at 3 months after curative-intent surgery. Patients were stratified according to an NLR cutoff of 2.5 (≤2.5 vs >2.5). Survival probabilities are shown for the entire cohort. The log-rank test demonstrated significantly reduced overall survival in patients with NLR > 2.5 (Log-rank p-value = 0.015). Numbers at risk are displayed below the plot.

**Figure 3:**
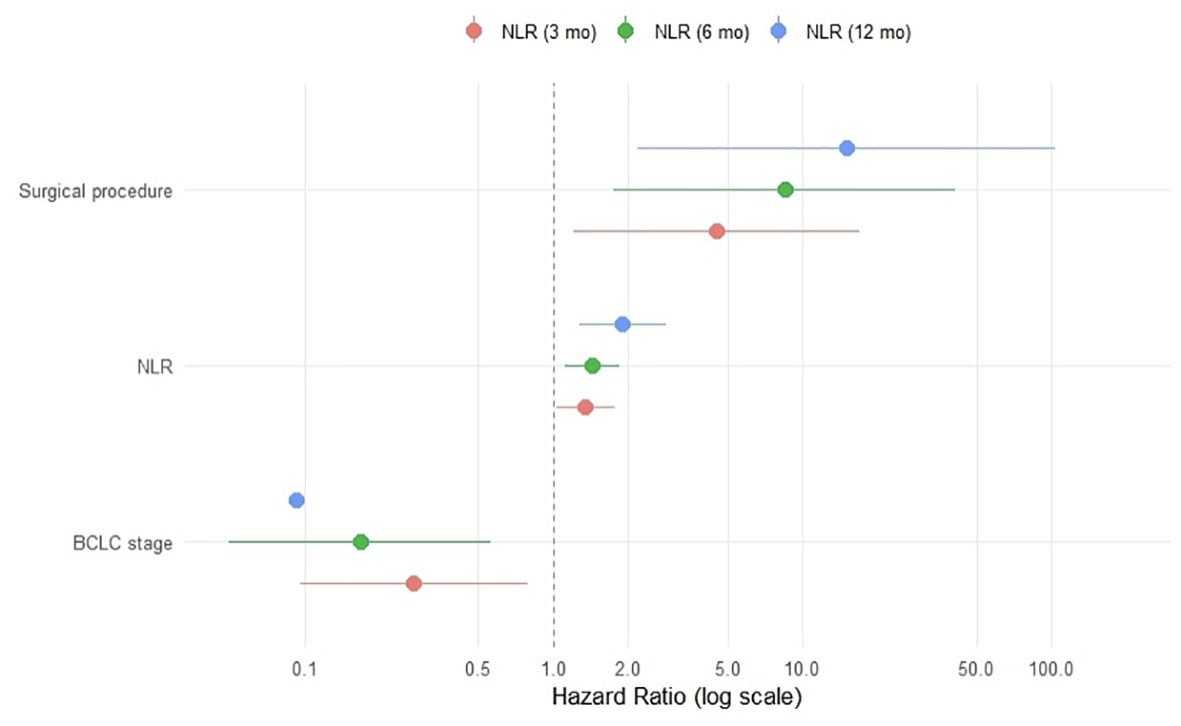
Forest plot of multivariable Cox proportional hazards regression models for OS. Hazard ratios (HRs) and 95% confidence intervals are shown for postoperative neutrophil-to-lymphocyte ratio (NLR) at 3, 6, and 12 months, surgical procedure (hepatectomy vs transplantation), and BCLC stage (0/A vs B/C). Models were adjusted for surgical modality and tumor stage.

Univariate Cox regression analyses evaluating the association of NLR and PLR at different postoperative time points with OS are summarized in **Table 2**. After a mean follow-up of 67.2 (95% CI: 58.0-69.0) months, the cumulative 1-, 3- and 5-year OS rates were 78.4%, 58.1%, and 40.5%, respectively. In univariate Cox regression models, higher NLR at 3, 6 and 12 months were significantly associated with worse OS (HRs: 1.509, 1.544, 1.349; p-values = 0.003, 0.001, 0.023, respectively). Additionally, PLR at 12 months was significantly associated with OS (HR 1.010, 95% CI: 1.003-1.018; p = 0.007, AICc = 75.67). Baseline and earlier postoperative PLR measurements did not demonstrate consistent prognostic value for OS. Multivariable Cox regression models adjusting for surgical procedure and BCLC stage are presented in **Table 3**. After adjustment for surgical procedure (hepatectomy vs transplantation) and BCLC stage (0/A vs B/C), NLR at 3 months (HR: 1.348; 95% CI: 1.024-1.773; p = 0.033), at 6 months (HR: 1.436; 95% CI: 1.115-1.848; p = 0.005) and at 12 months (HR: 1.900; 95% CI: 1.264-2.855; p = 0.002) remained an independent predictor of OS at all postoperative time points. Similarly, PLR at 12 months remained independently associated with OS (HR 1.008, 95% CI: 1.000-1.017; p = 0.037). Patients with an NLR > 2.5 at 3, 6, and 12 months experienced significantly worse OS, indicating its potential discriminatory capacity within this cohort for postoperative risk stratification. This difference in the OS according to the 3-month NLR cutoff is illustrated in **Figure 2**, while the independent prognostic effects and corresponding effect sizes are summarized in **Figure 3**.

**Table 2:** Univariate Cox proportional hazards regression analyses evaluating the association of NLR and PLR at predefined postoperative time points with OS and RFS.

Parameter	Univariate (OS)	Univariate (RFS)
NLR (baseline)		
HR	1.081	1.038
95% CI	0.976-1.197	0.947-1.137
p-value	0.136	0.423
-2 log likelihood	204.494	298.674
AICc	208.663	302.873
NLR (1 month)		
HR	1.016	0.891
95% CI	0.869-1.189	0.766-1.036
p-value	0.840	0.132
-2 log likelihood	121.374	212.893
AICc	125.543	217.062
NLR (3 month)		
HR	1.509	1.513
95% CI	1.148-1.983	1.136-2.013
p-value	**0.003**	**0.005**
-2 log likelihood	122.729	205.310
AICc	126.898	209.479
NLR (6 month)		
HR	1.544	1.249
95% CI	1.197-1.993	1.025-1.522
p-value	**0.001**	**0.028**
-2 log likelihood	100.101	188.387
AICc	104.270	192.556
NLR (12 month)		
HR	1.349	1.256
95% CI	1.043-1.745	1.021-1.544
p-value	**0.023**	**0.031**
-2 log likelihood	71.499	152.980
AICc	75.668	157.149
PLR (baseline)		
HR	1.002	1.000
95% CI	0.996-1.008	0.994-1.005
p-value	0.544	0.981
-2 log likelihood	204.494	298.674
AICc	208.663	302.873
PLR (1 month)		
HR	1.001	0.999
95% CI	0.997-1.004	0.996-1.002
p-value	0.683	0.593
-2 log likelihood	121.374	212.893
AICc	125.543	217.062
PLR (3 month)		
HR	1.005	1.002
95% CI	1.001-1.009	0.998-1.006
p-value	**0.026**	0.293
-2 log likelihood	122.729	205.310
AICc	126.898	209.479
PLR (6 month)		
HR	1.006	1.000
95% CI	0.996-1.016	0.992-1.008
p-value	0.219	0.992
-2 log likelihood	100.101	188.387
AICc	104.270	192.556
PLR (12 month)		
HR	1.010	1.010
95% CI	1.003-1.018	1.002-1.017
p-value	**0.007**	**0.018**
-2 log likelihood	71.499	152.980
AICc	75.668	157.149

HR, hazard ratio; OS, overall survival; RFS, recurrence-free survival; CI, confidence interval; NLR, neutrophil-to-lymphocyte ratio; PLR, platelet-to-lymphocyte ratio; AICc, corrected Akaike information criterion.

Bold values demonstrate statistical significance.

**Table 3:** Multivariable Cox proportional hazards regression models assessing the independent prognostic value of postoperative NLR and PLR for OS and RFS after adjustment for surgical procedure and BCLC stage.

Parameter	OS			RFS		
	NLR/PLR	Surgical procedure	BCLC stage	NLR/PLR	Surgical procedure	BCLC stage
NLR (baseline)						
HR	1.040	1.447	0.235	1.002	2.202	0.245
-95% CI	0.936	0.624	0.104	0.912	1.035	0.174
+95% CI	1.155	3.354	0.531	1.102	4.686	0.682
p-value	0.469	0.389	**0.001**	0.963	**0.040**	**0.002**
-2 log likelihood	204.494			298.674		
AICc	213.074			307.254		
NLR (1 month)						
HR	1.074	6.166	0.143	0.964	6.067	0.268
-95% CI	0.926	1.473	0.048	0.841	1.893	0.120
+95% CI	1.246	25.807	0.427	1.105	19.451	0.600
p-value	0.342	**0.013**	**<0.001**	0.596	**0.002**	**0.001**
-2 log likelihood	121.374			212.893		
AICc	129.924			221.473		
NLR (3 month)						
HR	1.348	4.549	0.276	1.402	6.102	0.413
-95% CI	1.024	1.214	0.096	1.063	1.989	0.178
+95% CI	1.773	17.052	0.793	1.850	18.726	0.961
p-value	**0.033**	**0.025**	**0.017**	**0.017**	**0.002**	**0.040**
-2 log likelihood	122.729			205.310		
AICc	131.309			213.890		
NLR (6 month)						
HR	1.436	8.500	0.168	1.159	11.850	0.366
-95% CI	1.115	1.746	0.050	0.966	3.214	0.153
+95% CI	1.848	41.381	0.562	1.390	43.690	0.875
p-value	**0.005**	**0.008**	**0.004**	0.112	**<0.001**	**0.024**
-2 log likelihood	100.101			188.387		
AICc	108.681			196.967		
NLR (12 month)						
HR	1.900	15.083	0.093	1.436	11.552	0.386
-95% CI	1.264	2.184	0.020	1.114	3.028	0.148
+95% CI	2.855	104.182	0.429	1.850	44.073	1.004
p-value	**0.002**	**0.006**	**0.002**	**0.005**	**<0.001**	0.051
-2 log likelihood	71.499			152.980		
AICc	80.079			161.560		
PLR (baseline)						
HR	0.999	1.455	0.221	0.997	2.396	0.315
-95% CI	0.992	0.624	0.098	0.991	1.111	0.160
+95% CI	1.006	3.393	0.498	1.003	5.168	0.621
p-value	0.845	0.386	**<0.001**	0.300	**0.026**	**0.001**
-2 log likelihood	204.494			298.674		
AICc	213.074			307.254		
PLR (1 month)						
HR	1.001	5.241	0.140	0.999	6.336	0.268
-95% CI	0.997	1.224	0.047	0.996	2.002	0.118
+95% CI	1.004	22.432	0.421	1.003	20.053	0.607
p-value	0.744	**0.026**	**<0.001**	0.754	**0.002**	**0.002**
-2 log likelihood	121.374			212.893		
AICc	129.954			221.473		
PLR (3 month)						
HR	1.002	4.252	0.237	1.000	6.039	0.339
-95% CI	0.999	1.156	0.086	0.997	1.983	0.151
+95% CI	1.006	15.637	0.652	1.004	18.390	0.761
p-value	0.225	**0.029**	**0.005**	0.902	**0.002**	**0.009**
-2 log likelihood	122.729			205.310		
AICc	131.309			213.890		
PLR (6 month)						
HR	1.010	10.971	0.145	1.001	12.070	0.328
-95% CI	1.000	2.153	0.044	0.993	3.273	0.141
+95% CI	1.020	55.898	0.478	1.008	44.509	0.763
p-value	0.059	**0.004**	**0.002**	0.858	**<0.001**	**0.010**
-2 log likelihood	100.101					
AICc	108.681					
PLR (12 month)						
HR	1.008	7.658	0.127	1.007	8.865	0.387
-95% CI	1.000	1.525	0.031	1.000	2.446	0.147
+95% CI	1.017	38.442	0.520	1.015	32.138	1.022
p-value	**0.037**	**0.013**	**0.004**	0.055	**0.001**	0.055
-2 log likelihood	71.499			152.980		
AICc	80.079			161.560		

NLR/PLR values, surgical procedure (1: hepatectomy; 0: transplantation) and BCLC stage (1: BCLC stage 0/A; 0: BCLC stage B/C) were considered as independent variables. HR, hazard ratio; OS, overall survival; RFS, recurrence-free survival; CI, confidence interval; NLR, neutrophil-to-lymphocyte ratio; PLR, platelet-to-lymphocyte ratio; BCLC, Barcelona Clinic Liver Cancer; AICc, corrected Akaike information criterion.

Bold values demonstrate statistical significance.

To mitigate potential immortal-time bias because postoperative NLR is only measurable in patients surviving to the assessment timepoint, a 3-month landmark analysis was performed, restricting the cohort to patients alive and event-free at 3 months and measuring subsequent outcomes from that landmark. In this landmark analysis, higher 3-month NLR remained associated with poorer OS (HR 1.621; 95% CI 1.076-2.442; p = 0.021), whereas the association with RFS did not reach statistical significance (HR 1.347; 95% CI 0.956-1.898; p = 0.089). In sensitivity analyses restricted to hepatectomy patients and additionally adjusting for vascular invasion, nodal spread, or prior transarterial chemoembolization (TACE), the association between 3-month NLR and OS remained directionally consistent (NLR HRs: 1.356 [p = 0.033], 1.338 [p = 0.045], and 1.347 [p = 0.033], respectively), while the added covariates were not independently associated with OS (vascular invasion HR 1.204, p = 0.752; nodal spread HR 1.123, p = 0.863; TACE HR 1.033, p = 0.961).

### 3.5 Recurrence-free survival analysis

**Figure 4:**
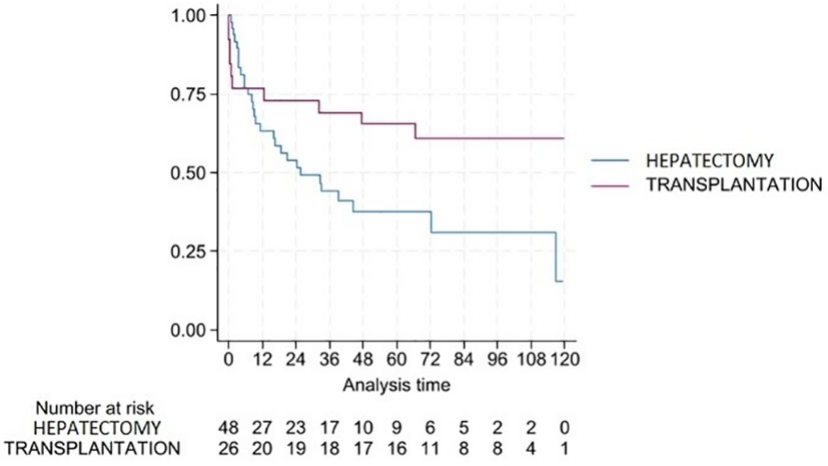
Kaplan-Meier curves for recurrence-free survival (RFS) stratified by surgical modality. Patients undergoing hepatectomy were compared with those undergoing orthotopic liver transplantation. Hepatectomy was associated with significantly shorter recurrence-free survival (Log-rank p-value = 0.051). Numbers at risk are displayed below the plot.

Recurrence-free survival differed markedly according to surgical modality, reflecting expected differences in baseline liver function, tumor characteristics, and selection; accordingly, surgical modality was treated as an adjustment covariate and no causal comparison was intended. Mean RFS was 58.3 months (95% CI: 41.8-74.8) following hepatectomy and 117.7 months (95% CI: 108.6-126.8) following transplantation (p < 0.001). Multivariable Cox regression analyses for RFS, incorporating inflammatory indices, surgical modality, and BCLC stage, are detailed in **Table 3**. In the multivariate model, independent predictors of reduced RFS included surgical modality (hepatectomy compared to OLT; HR range 6.10-11.85; all p ≤ 0.002), elevated NLR at 3 (HR 1.402 95% CI: 1.063-1.850, p = 0.017) and 12 months (HR 1.436 95% CI: 1.114-1.850, p = 0.005), while PLR showed a time-dependent effect, with PLR at 12 months demonstrating borderline to significant prognostic value for RFS (HR 1.007 95% CI: 1.000-1.015, p = 0.055). Patients with NLR > 2.5 at 3 or 12 months had significantly shorter RFS (p = 0.003), confirming the discriminatory capacity of this cutoff for recurrence risk. Kaplan-Meier curves depicting RFS according to surgical modality are shown in **Figure 4**. Patients with an NLR exceeding 2.5 at 3 or 12 months had significantly shorter RFS (p = 0.003). In contrast, no significant associations between NLR/PLR and RFS were observed in the OLT subgroup. Results of univariate Cox regression analyses assessing the prognostic impact of postoperative NLR and PLR on RFS are demonstrated in **Table 2**.

## 4 Discussion

In this multicenter retrospective study, we evaluated the prognostic role of systemic inflammatory indices in patients with HCC undergoing curative-intent liver resection or orthotopic liver transplantation. By incorporating serial postoperative measurements and time-varying Cox regression models, we demonstrate that the NLR is a robust, time-independent predictor of overall survival, whereas its association with recurrence-free survival is not sustained longitudinally. These findings refine the role of inflammation-based biomarkers in surgically treated HCC. A key observation is that NLR retains statistically significant prognostic value for OS independently of surgical modality, BCLC stage, and timing of measurement. This suggests that NLR reflects a persistent systemic inflammatory and immunological state rather than a transient perioperative response, while the absence of a significant interaction between NLR and time further supports the stability of its effect. The discriminatory performance of the 3-month NLR (C-index 0.79) indicates good predictive accuracy for survival outcomes, consistent with prior studies evaluating inflammatory biomarkers in HCC and other solid tumors, although direct comparisons should be interpreted cautiously (23–25). Thus, given its low cost, reproducibility, and availability, NLR represents a practical adjunct for postoperative risk stratification.

From a biological perspective, elevated NLR reflects a complex interplay between tumor-promoting inflammation and impaired antitumor immune surveillance (26). Neutrophils contribute to tumor progression through cytokine release, angiogenesis, and facilitation of tumor invasion and metastasis (13). Mechanistically, neutrophil extracellular traps (NETs) promote metastatic dissemination and immune evasion, while myeloid-derived suppressor cells (MDSCs) suppress T-cell-mediated antitumor immunity, contributing to an immunosuppressive tumor microenvironment (27). Conversely, lymphocytes are essential for immune surveillance, and lymphopenia reflects impaired host defense (16). Therefore, elevated NLR should be interpreted not merely as a numerical ratio but as a surrogate marker of systemic immune dysregulation and imbalance between tumor-promoting inflammation and host immune defense. These mechanisms provide a biologically plausible explanation for the association between elevated postoperative NLR and reduced overall survival, supporting the role of persistent inflammation and immune dysfunction in adverse HCC outcomes. Persistent postoperative elevation of NLR may reflect ongoing immune dysregulation, chronic liver inflammation, or systemic vulnerability, all of which may negatively impact long-term survival even after curative treatment. Recent evidence further refines this concept by distinguishing functional phenotypes of tumor-associated neutrophils. Neutrophils may polarize into antitumor (N1) or protumor (N2) subsets. N1 neutrophils exhibit cytotoxic and immunostimulatory activity, whereas N2 neutrophils promote tumor growth, angiogenesis, and immune suppression (13). In HCC, the tumor microenvironment-particularly via cytokines such as transforming growth factor-β (TGF-β) favors polarization toward the N2 phenotype, contributing to disease progression (14). This functional plasticity provides a mechanistic framework for interpreting elevated NLR, as it likely reflects a predominance of protumor neutrophil activity, while emerging research highlights neutrophil polarization as a dynamic and potentially targetable component of tumor immunobiology, further supporting the clinical relevance of inflammation-based biomarkers (26, 27).

In contrast to its consistent association with OS, NLR did not demonstrate stable longitudinal prognostic significance for RFS when modeled dynamically. Although associations were observed at specific postoperative time points, these were not sustained in time-varying analyses. This suggests that NLR reflects survival vulnerability rather than recurrence biology. Recurrence in HCC is primarily driven by tumor-related factors and the carcinogenic liver microenvironment, whereas systemic inflammation and immune competence more strongly influence survival following recurrence or competing mortality risks (14, 16). This distinction is clinically relevant, indicating that NLR is more suitable for postoperative risk stratification than for predicting recurrence. The prognostic relevance of NLR was primarily observed in patients undergoing hepatectomy, whereas no significant associations were detected in the transplantation subgroup. This likely reflects biological differences between the two settings, with hepatectomy preserving the chronically inflamed liver, allowing ongoing inflammatory processes to influence outcomes (28). In contrast, transplantation removes both tumor and diseased liver and introduces immunosuppressive therapy, which may obscure the prognostic signal of inflammatory biomarkers (29). On the other hand, PLR demonstrated more limited and time-dependent prognostic value. Preoperative PLR predicted major postoperative complications, while elevated PLR at 12 months was associated with reduced OS and, to a lesser extent, RFS. Platelets are increasingly recognized as active contributors to tumor progression through angiogenesis, immune evasion, and protection of circulating tumor cells (30, 31), which may explain the delayed prognostic effect observed. Clinically, these findings support the use of postoperative NLR-particularly at approximately 3 months-as an adjunct for risk stratification, while patients with persistently elevated NLR may represent a higher-risk subgroup that could benefit from closer surveillance strategies, with NLR complementing, not replacing, established clinicopathological factors.

This study has several important strengths addressing gaps in the literature on inflammation-based biomarkers in HCC. The multicenter design and inclusion of patients from high-volume tertiary and transplantation centers enhance cohort representativeness and reduce center-specific bias. Inclusion of both liver resection and orthotopic liver transplantation reflects real-world therapeutic heterogeneity and enables evaluation across the two principal curative strategies. A major methodological strength is the longitudinal assessment of NLR and PLR, with serial measurements up to 12 months, overcoming limitations of single time-point analyses that may reflect transient perioperative inflammation rather than true systemic immune states. The use of time-varying Cox models enables dynamic evaluation of biomarker effects over time while avoiding arbitrary measurement windows. The persistence of NLR as a predictor of OS within this framework supports its biological relevance. Model discrimination was assessed using Harrell’s concordance statistic, a survival-specific metric relevant for clinical evaluation. The observed C-index approaching 0.80 indicates meaningful predictive performance and supports the prognostic value of NLR beyond statistical association, consistent with prior studies, although comparisons should be interpreted cautiously. Adjustment for key confounders, including surgical modality and BCLC stage, further supports that NLR provides prognostic information independent of disease stage and treatment allocation.

However, several limitations warrant consideration. The retrospective design introduces potential selection and information biases, although this is common in exploratory biomarker studies with longitudinal postoperative data, particularly in transplantation cohorts. Exclusion of 30-day postoperative mortality was applied to reduce bias from perioperative events unrelated to tumor biology, strengthening the validity of survival analyses. The moderate sample size, especially in the transplantation subgroup, limits statistical power for subgroup analyses and interaction testing. Internal validation using bootstrap resampling (1,000 iterations) partially mitigates this concern and supports model robustness. The absence of prognostic associations in transplanted patients is biologically plausible, reflecting immunologic and inflammatory alterations induced by liver replacement and immunosuppressive therapy rather than insufficient power alone; thus, findings should be considered hypothesis-generating. NLR and PLR are non-specific markers of systemic inflammation and may be influenced by infections, comorbidities, or medications, although this was partly mitigated through serial measurements and dynamic modeling. The proposed NLR cutoff (>2.5), derived from 3-month postoperative values, demonstrated discriminatory capacity but remains data-driven and exploratory, requiring external validation. Although a potential role of NLR in post-recurrence survival is suggested, this was not directly assessed and requires further study. An additional limitation is the absence of formal competing-risk analysis, as non-recurrence-related death may influence RFS estimates, particularly given differing mortality profiles between hepatectomy and transplantation. Finally, although a 5-year survival-based NLR cutoff was proposed and internally validated, external validation in larger prospective cohorts is required before clinical implementation. Despite these limitations, the study’s methodological rigor, biologically coherent findings, and advanced modeling support its conclusions and justify further prospective validation of NLR as a prognostic biomarker in HCC.

The present findings, demonstrating a stable and time-independent association between postoperative NLR and OS-particularly following liver resection, provide a framework for future research in HCC. Prospective multicenter studies with standardized postoperative sampling are required to validate NLR as a reproducible biomarker and confirm the clinical applicability of a 5-year survival-based cutoff across populations. Future work should focus on integrating NLR into postoperative prognostic algorithms alongside established tumor- and liver-related factors, rather than evaluating it in isolation. Such composite models may improve identification of patients at increased mortality risk after curative surgery, particularly in the hepatectomy setting where persistent inflammation influences outcomes (32). Mechanistic studies are warranted to clarify the biological basis of persistently elevated NLR after surgery. Elucidating the roles of neutrophil-driven inflammation, immune suppression, and lymphocyte dysfunction may help distinguish sustained immune dysregulation from transient responses and inform targeted strategies. Clinically, patients with persistently elevated postoperative NLR may benefit from closer surveillance, such as shorter imaging intervals or intensified follow-up, although NLR should not be used in isolation and must be interpreted alongside established clinicopathological factors. As systemic and immunomodulatory therapies are increasingly introduced earlier in HCC management (33), future studies should evaluate whether NLR can serve not only as a prognostic marker but also as a tool to guide surveillance or treatment strategies. Given its simplicity, low cost, and wide availability, NLR represents a pragmatic candidate biomarker for prospective trials and real-world application.

In conclusion, this multicenter study demonstrates that NLR is a time-independent predictor of OS in patients with HCC undergoing curative-intent surgery, particularly following liver resection. In contrast, NLR does not show consistent longitudinal prognostic value for RFS, supporting its role as a marker of survival vulnerability rather than recurrence biology. PLR provides complementary information, particularly regarding perioperative risk and late outcomes. These findings support the integration of simple inflammation-based biomarkers into postoperative prognostic assessment in HCC and justify their further evaluation in prospective studies.

## Data Availability

The raw data supporting the conclusions of this article will be made available by the authors, without undue reservation.
